# New Insights on the Spin Glass Behavior in Ferrites Nanoparticles

**DOI:** 10.3390/nano12101782

**Published:** 2022-05-23

**Authors:** Emil Burzo, Romulus Tetean

**Affiliations:** Faculty of Physics, “Babes Bolyai” University, Kogalniceanu 1, 400084 Cluj-Napoca, Romania

**Keywords:** ferrite nanoparticles, magnetic properties, spin-glass, exchange field

## Abstract

The magnetic properties of nanocrystalline M_x_Fe_3−x_O_4_ ferrites with M=Fe, Co, and Zn were investigated. The data support a core–shell model, where the core is ferrimagnetically ordered, and the shell shows a spin glass type behavior. The reduced magnetizations of spin glass components follow an m_g_ = (1 – *b/H*^−1/2^) field dependence. The b values are strongly correlated with the intensities of exchange interactions. The field dependences of the magnetoresistances of Fe_3_O_4_ and Zn_x_Fe_3−x_O_4_ nanoparticles pellets, experimentally determined, are well described if instead of the core reduced magnetization, commonly used, that of the shell is taken into account. For similar compositions of the nanoparticles, identical *b* values are obtained both from magnetization isotherms and magnetoresistances studies. The half-metallic behavior of spinel Fe_3_O_4_ based nanoparticles is discussed comparatively with those of double perovskites.

## 1. Introduction

Ferrite nanoparticles with a spinel-type structure have garnered a great deal of attention due to their basic properties and applications in various fields such as medicine [1], adsorption potential to abate heavy metals and dyes from aqueous solutions [2], catalytic properties [3], magnetoresistive devices [4], etc. The Fe_3_O_4_ based nanoparticles with spinel-type structure can be described as core–shell systems, where the structure and magnetic properties of the shell are different from that of the core [5].

The bulk magnetite (Fe^3+^)_A_[Fe^3+^Fe^2+^]_B_, at ambient temperature, has a cubic inverse spinel-type structure. In this lattice, the O^2−^ anions form an *fcc* type lattice, the Fe^3+^ ions being located in tetrahedral interstices (A) and the Fe^3+^ and Fe^2+^ in the octahedral interstices (B). The nature of spinel structures, such as normal, inverse, or mixed in substitutional ferrites Fe_3−x_M_x_O_4_ with M=Co or Zn, relies on lattice occupancy by these ions. The Zn^2+^ ions are mainly located in tetrahedral sites [6,7]. The cobalt ferrites exist as partially inverse spinel structures in which both A and B sites contain a fraction of Co^2+^ ions, the largest being located at B sites [5,8].

Upon cooling, bulk Fe_3_O_4_ displays a sharp Verwey transition at the temperature *T*_V_ = 122 K, characterized by a structural transition from a cubic to monoclinic lattice together with an abrupt drop in the electrical conductivity, associated with a “freezing out” of the electron hopping between the Fe^2+^ and Fe^3+^ ions in B sublattice, which is the primary conduction mechanism at temperatures *T* > *T*_V_ [9].

The surface structure of magnetite differs from that of bulk material. A large number of studies were performed in order to analyze the Fe_3_O_4_ surfaces. There are two possible truncations [10]. At the Fe_3_O_4_ (111) surface, three distinct terminations are observed, exposing either a close-packed oxygen plane, Fe_A,_ or Fe_B_ atoms [11]. The stable Fe_3_O_4_ (111) termination might have oxygen [12,13] or contain a fraction of iron and oxygen monolayers exposed over a closely packed oxygen layer [14,15]. In the case of Fe_3_O_4_ (100), surface terminations with ordered oxygen vacancies or Fe adatoms were proposed [16]. The surface structures are dependent on the sample’s preparation conditions, and thus multiple terminations can exist concurrently [13]. The oxygen termination has been shown to be inert toward adsorbate, whereas cation terminations introduce reactivity [17,18].

Magnetite is ferrimagnetically ordered with magnetic moments of Fe_A_ and Fe_B_ sites antiparallel oriented. In bulk Fe_3_O_4_, there are four easy magnetization [111] axes above the Verwey temperature *T*_V_. For Fe_3_O_4_ [111] surfaces, one of the axes is perpendicular to the [111] surface, and the other three make an angle of 109.5° with respect to the surface’s normal direction [19].

The surface structures in nanocrystalline ferrites influence their magnetic properties because of symmetry breaking. The magnetic properties of bulk samples are little influenced by surface effects, the surface volume being only a very small fraction of the sample. In nanocrystalline samples, the surface volume represents a large fraction of that of the nanoparticles. The reduction of saturation magnetization as compared to bulk values is a common experimental observation in magnetite nanoparticles [20]. In early models, this behavior was attributed to the presence of a dead magnetic layer at the surface [21]. A random canting of the surface spins caused by competing antiferromagnetic interactions between sublattices was proposed [22] and experimentally observed in maghemites [23,24,25]. A spin glass type behavior was also shown in the whole volume of nanoparticles [26,27]. A model of a magnetically ordered core surrounded by a surface layer of canted spins has been also proposed [28,29]. The reduction of *T*_C_ of nanoparticles with respect to the bulk one was also attributed to symmetry breaking of the surface and consequently to a lower density of magnetic bonds [30]. The noncollinear spin structure, which originated from the pinning of the surface spins and coated surfactant at the interface of iron oxide results in the reduction of magnetic moments in nanoparticles [31].

Surfactant organic molecules, such as oleic acid, can restore the magnetism in Fe_3_O_4_ nanoparticle surfaces [32]. Of the four Fe ions at the surface unit cell, two bond to the organic acid, whereas the other two remain unbonded. The Fe ions bonded to the organic acid oxygens have six O nearest neighbors as in the bulk, while the remaining iron ions are similar to the bare surface. The overall effect is that capped surface magnetization density is intermediate between that of bulk and the magnetic layer surface of the bare nanoparticle. The formation of the half-metallic surface state for pyridine/H/Fe_3_O_4_ nanoparticles can also be understood on the basis of the interface chemical bonding formed by the coordination of the nitrogen end of pyridine to the surface of Fe atoms [33].

In nanocrystalline Fe_3_O_4_, the spin canting effect can be induced by: (1) the symmetry breaking by the broken exchange bonds at the surface layer; (2) competition between the ferromagnetic interactions inside the magnetic sublattices and antiferromagnetic between them; (3) the cations distribution in tetrahedral and octahedral sites; (4) the surface anisotropy which depends on the iron site occupation.

In this paper, we analyze the magnetic behavior of some nanocrystalline iron-based ferrites by extending our previous studies [5,7,34]. The presence of spin-glass behavior superposed on essentially ferrimagnetic-type ordering was shown and analyzed in correlation with the exchange interactions between the two sublattices. The field dependences of the magnetoresistances are well described when using instead of core reduced magnetization that of nanoparticles shell, of spin-glass type, highlighting their importance in magnetotransport properties. These properties of spinelic ferrites are analyzed comparatively with those of double perovskites.

## 2. Materials and Methods

**Figure 1:**
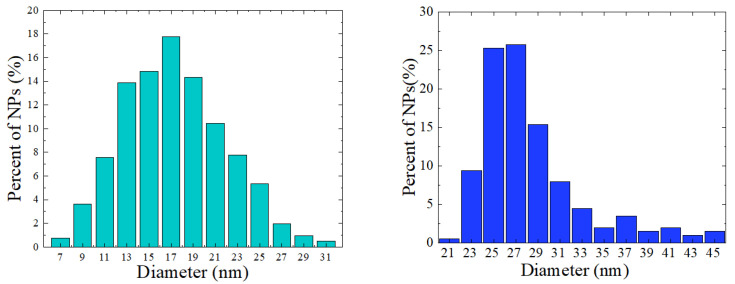
The size distributions for: Fe_3_O_4_ (**left**) and Zn_0.12_Fe_2.88_O_4_ (**right**) nanoparticles.

The nanocrystalline ferrites from series Fe_3_O_4_, CoFe_2_O_4,_ and Zn_x_Fe_3−x_O_4_ were prepared using a typical hydrothermal method, as already described [5,7,34]. The morphology of the nanoparticles has been investigated by transmission electron microscopy (TEM) and scanning electron microscopy (SEM) using Hitachi HD2700 equipment. The compositions of the nanoparticles were determined by the EDS method and by chemical analyses. The elemental analyses by the ICP-OES method yielded, for Zn_x_Fe_3−x_O_4_ nanoparticles, values of x = 0.12(3) and 0.18(3), respectively, in rather good agreement with EDS measurements. The crystal structure and mean crystallite sizes were determined by XRD measurements, performed at ambient temperature with a Bruker DS Advance diffractometer. The mean crystallite sizes were estimated by Rietveld refinement of the XRD patterns using FullProf Suite software. The investigated nanocrystalline ferrites crystallize in a cubic type spinel structure with lattice parameters given in Table 1. No other phases were present in their XRD patterns. The nanocrystallite sizes were determined by the analysis of their histograms as given in Figure 1 for Fe_3_O_4_ and Zn_0.12_Fe_2.88_O_4_ samples. The mean nanocrystalline sizes are in closer agreement with those estimated from X-ray measurements. The data for CoFe_2_O_4_ nanoparticles were already reported [5]. The determined lattice parameters and mean nanograins sizes are listed in Table 1.

Magnetic measurements were made at *T* = 4.2 K and 300 K in external fields up to 12 T using a vibrating sample magnetometer from Cryogenic Limited (London). In order to obtain accurate values of the magnetizations, at *T* = 4.2 K, attention has been given to stabilizing the external field.

## 3. Results

**Figure 2:**
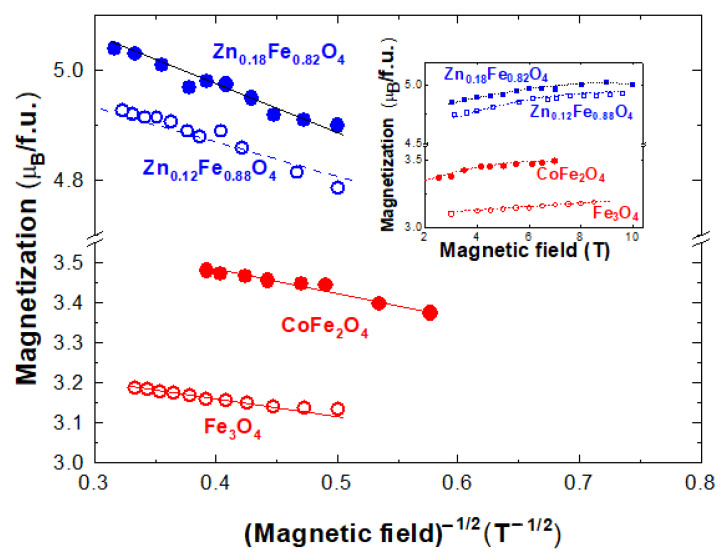
Field dependences of magnetizations at *T* = 4.2 K in Fe_3_O_4_, CoFe_2_O_4_ and Zn_x_Fe_3−x_O_4_ with x = 0.12 and 0.18 ferrites. In the inset are the respective magnetization curves.

The magnetization isotherms at *T* = 4.2 K for selected Fe_3_O_4_ and Fe_3−x_M_x_O_4_ with M=Co and Zn nanoparticles are given in Figure 2 (inset). Their saturation magnetizations are somewhat lower than those of the bulk samples having the same compositions [35,36]. This trend was attributed to the small particle size effect, where noncollinear spin arrangements occur primarily at or near the surface. Close related data were obtained in the Fe_3−x_Zn_x_O_4_ nanoparticles system [7,37,38,39].

The field dependences of magnetization for a spin glass system are determined by the anisotropy as well as on the exchange field, *H*_ex,_ acting on magnetic ions, as also evidenced in amorphous systems [40]. When the anisotropy is weak (ferromagnet with wondering axes), the approach to saturation when the external field, *H*, is smaller than the exchange field, *H_ex_,* can be described by a 1/*H*^−1/2^ law, while for *H* > *H_ex_* follows a 1/*H*^2^ trend as for systems having high anisotropy. Consequently, both the anisotropy and exchange fields in the investigated systems were estimated in order to correlate with model prediction.

The estimated anisotropy constants are rather low in the order of (1–4)10^4^ J/m^3^. The exchange fields, *H_ex_* in the studied systems were estimated from the exchange interaction parameters *J_AB_* between the two sublattices, in the mean-field approximation:

(1) $$H_{ex}=J_{AB}Szi/g\mu_{0}\mu_{B}$$

where *S* is the spin value, *z*_i_ the number of magnetic nearest neighbors, *g* the spectroscopic splitting factor, and *µ_B_* the Bohr magneton.

The exchange interactions *J_AB_*, between the two magnetic sublattices determined by neutron diffraction are *J_AB_* = −2.02 meV for Fe_3_O_4_ and −1.95 meV for CoFe_2_O_4_ [41]. The exchange interactions between iron ions in tetrahedral and octahedral sites, respectively, in Zn_x_Fe_3−x_O_4_ were estimated starting from magnetic measurements [7,41,42] Values *J_AB_* = 1.64 meV for x = 0.18 and 1.76 meV when x = 0.12 were obtained.

The Zn_x_Fe_3−x_O_4_ system has very interesting magnetic properties. The ferrimagnetic phase coexists with antiferromagnetic and spin disordered regions [43]. The above behavior can be correlated with the presence of iron ions having different local environments, where the number of non-magnetic Zn ions predominates. Consequently, the exchange interactions between iron ions are rather low, and the spin disorder disappears even in the presence of a low magnetic field. When iron is substituted by a small fraction of Zn ions, as in the Zn_x_Fe_3−x_O_4_ series with x ≤ 0.18, the exchange interactions both inside and between magnetic sublattices are rather strong and the samples remain ferrimagnetically ordered. The magnetic coupling between octahedral and tetrahedral sublattices decreases only by ≅19% when x = 0.18 as compared to that in pure Fe_3_O_4_.

The location of Zn ions in the Zn_x_Fe_8-x_O_4_ nanoparticles systems was determined from magnetic measurements, the extrapolated moment at *T* = 4.2 K, and *H*→∞, respectively. In this state, the iron magnetic moments are oriented along the same axis in the framework of ferrimagnetic ordering. The highest magnetization for x < 0.3 is obtained when Zn^2+^ ions are distributed in tetrahedral sites. The expected magnetic moments for this location are 4.72 µ_B_/f.u. when x = 0.12 and 5.08 µ_B_/f.u. for x = 0.18, respectively. The experimentally determined saturation moments are ≅5% higher than the above values suggesting that Zn ions occupy the tetrahedral sites. The observed differences can be correlated with the sample’s compositions situated within the low limit of experimental errors.

Taking into account the distributions of constituent ions in tetrahedral and octahedral sites [5,7,34], the exchange fields, *H_ex_*, acting on octahedral and tetrahedral sites, were estimated in the mean-field approximation, according to relation (1). In the spinel structure, as Fe_3_O_4_, each tetrahedral Fe^3+^ ion is surrounded by 12 octahedral ions, while an octahedral Fe^3+^ has 6 tetrahedral nearest neighbors. According to the distributions of ions in [Co^2+^_0.838_Fe^3+^_1.162_](Co^2+^_0.162_ Fe^3+^_0.838_)O_4,_ a tetrahedral Fe^3+^ ion, has as neighbors 4 Fe and 2 Co octahedral ions and an octahedral Fe^3+^ has 7 Co and 5 Fe tetrahedral ions, respectively [4]. In [Fe^3+^_1.12_ Fe^2+^_0.88_](Fe^3+^_0.88_Zn^2+^_0.12_)O_4_ in tetrahedral sites are located in mean 5 Fe^3+^ and 1 Zn^2+^ ions. On this basis, the exchange fields acting on iron in octahedral and tetrahedral sites were estimated. As for example in CoFe_2_O_4_, these values were estimated at *H_ex_*(oct) = 110 T and *H_ex_*(tetra) = 55 T, respectively. Somewhat smaller values of 90 T and 38 T were obtained in Zn_0.12_Fe_2.88_O_4_ ferrite. Although these values characterize the bulk samples, they give a rather good approximation in the case of nanocrystalline systems. The exchange field acting on magnetic ions in octahedral sites is higher than in tetrahedral ones, and both are higher than the external field used for measurements. Consequently, a field dependence of *H*^−1/2^ is suggested. In addition, at the nanograin surface, different spin canting for octahedral and tetrahedral ions is expected, in addition to the effect of broken bonds.

**Figure 3:**
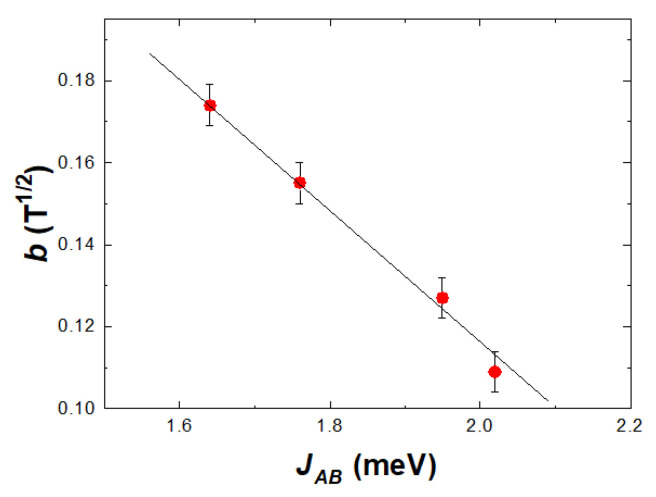
The b parameter as a function of exchange interactions between magnetic sublattices.

The magnetic properties of these nanoparticles can be described as a superposition of a spin glass contribution on mainly ferrimagnetic type ordering. Spin glasses are a highly complex magnetic state intrinsically linked to spin frustration and structural disorder. For external fields greater than 3–4 T, when the core particle magnetization is saturated, the magnetization isotherms follow a field dependence described by the relation:

(2) $$\frac{M\left(H\right)}{M\left(0\right)}=m_{g}\left(H\right)=1-b/H^{-1/2}$$

as predicted by the model [40] in Figure 1. The determined b parameters, at *T* = 4.2 K, describe the approach to saturation behavior of the spin-glass component. These values increase in the sequence *b* = 0.108 T^−1/2^ for magnetite, 0.127 T^−1/2^, for cobalt ferrite, while for Zn_x_Fe_3−x_O_4_, *b* = 0.155 T^−1/2^ for x = 0.12 and 0.174 T^−1/2^ when x = 0.18. The *b* values, determined at higher temperatures, are a little smaller than those obtained at *T* = 4.2 K, a behavior attributed to thermal effects. A linear dependence of *b* parameters on the *J_AB_* values is shown in Figure 3. These data suggest that the approach to saturation of spin glass components (parallel alignment of the spins) is more difficult as the exchange interactions between the two sublattices increase. It is to be noted that in CoFe_2_O_4_ nanoparticles, there is a change of spin-glass behavior in fields higher than 8–9 T, correlated with the presence of magnetic Co ions [5]. The extrapolation of magnetization at *T* = 4.2 K according to 1/*H*^−1/2^ law, to infinite fields is expected to characterize the situation when the moments at both core and shell are oriented along the same axis and are ferrimagnetically ordered.

Assuming core–shell sphere type nanoparticles, the relative volume corresponding to a shell having the width of one lattice parameter is dependent on the nanograins diameter, being 15% for the Zn_0.12_Fe_2.88_O_4_ and 27–31% for Fe_3_O_4_, CoFe_2_O_4,_ and Zn_0.18_Fe_2.82_O_4_ nanoparticles. The spin-glass contribution to the magnetization was estimated as the difference between magnetizations obtained by extrapolation of *H*^−1/2^ dependence at infinite field and saturation magnetizations by using the classical approach to saturation law. These are between 7% and 9% of the total magnetizations. This suggests that not all the magnetic ions from one atomic unit cell are involved in the spin-glass type magnetism. This is in agreement with the result of Fe_3_O_4_ interface studies, where only a fraction of magnetic ions from the unit cell have surface terminations and breaking bonds [14,15] and thus spin-glass type magnetism.

The bulk spin-resolved band structure of Fe_3_O_4_ predicts that the majority spin population is insulating in character and the minority carriers possess a metallic character, with states derived predominantly from the Fe3*d* bands of octahedral sublattice, present at the Fermi level [44,45]. Due to their half-metallic properties and high Curie temperature, these ferrites are of interest for spintronic applications [33,46]. Magnetite was assumed to be a candidate for building sensors based on the intergrain tunneling magnetoresistance (ITMR) at ambient temperature. As already discussed, the Fe_3_O_4_ core–shell nanoparticles have a core material with high spin polarization and a shell that has a spin glass oxygen termination, which can act as an insulator.

There are a large number of studies concerning the analysis of the magnetoresistances (*MR*) of the Fe_3_O_4_ nanoparticles pressed into pellets on the external field, temperature, grain sizes, or their shape [47,48,49,50,51,52,53,54]. Magnetoelectronic devices composed of ordered three-dimensional arrays of magnetite nanoparticles on the SiO_2_ isolation layer [51] as well as of the SiO_2_ coated Fe_3_O_4_ nanospheres were investigated [55,56]. The magnetoresistances of Fe_3_O_4_-CoFe_2_O_4_ core–shell nanoparticles [57] were also studied.

The magnetoresistances in Fe_3_O_4_ were investigated above the Verwey temperature. The *MR* was shown to be of tunneling type [4,50,52,54,58]. The field dependences of the magnetoresistance Δ*ρ*/*ρ* were also analyzed [55,59] starting from the relation [60]:

(3) $$\frac{\Delta \rho }{\rho }=-P^{2}{\left[m\left(H\right)\right]}^{2}/(1+P^{2}{\left[m\left(H\right)\right]}^{2})$$

where *P* is the polarization and *m* is the reduced bulk (core) magnetization.

The above relation does not describe well the field dependence of magnetoresistances. The Ziese model [60] was used in order to analyze the spin polarization in Fe_3_O_4_ nanorods [53]. A difference in the saturation fields of sample magnetization and magnetoresistance was also shown [51].

The ITMR in polycrystalline magnetic materials is determined by the magnetic state in the vicinity of the grain boundary (GB) [50,61,62,63]. Thus, in relation (3), the reduced bulk magnetization *m*(*H*) must be replaced by that at the grain boundary *m_g_*(*H*), as given by the relation (1). The study of Fe_3_O_4_ nanograins magnetoresistance in high external fields also evidenced a contribution due to spin disorder, which is linear in the field [61]. Taking the above into account, the relation (3) used to analyze in order to analyze the experimental data has the form [62,63]:

(4) $$\frac{\Delta \rho }{\rho }=-\frac{P^{2}{\left[m_{g}\left(H\right)\right]}^{2}}{\left(1+P^{2}{\left[m_{g}\left(H\right)\right]}^{2}\right)}+cH$$

**Figure 4:**
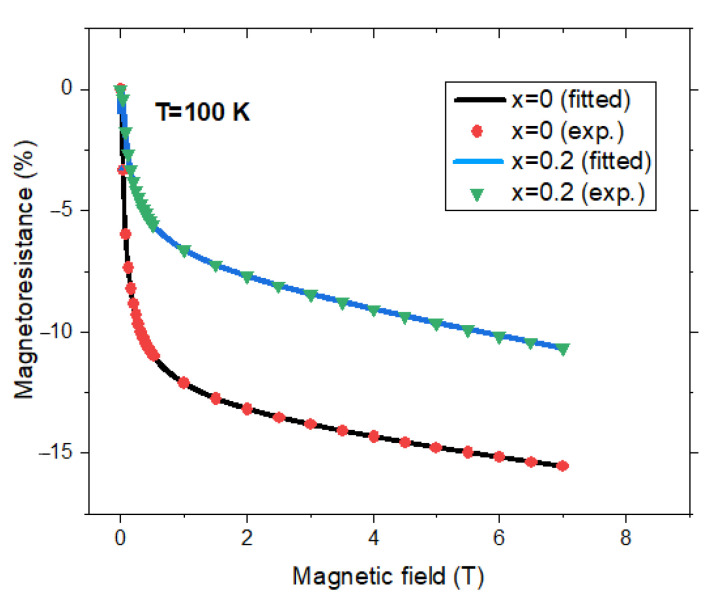
Field dependences of magnetoresistances for Zn_x_Fe_3−x_O_4_ nanocrystalline pellets with x = 0 and 0.2. Experimental data [38] and fitted curves according to relation (4) with parameters listed in Table 2.

By fitting experimental data, (for fields higher than 0.1 T (where *MR* were estimated with accuracy) [33,38,46,50,51,54,58], with the relation (4), the *P*, *b,* and *c* values were obtained, see Table 1. The curves thus obtained describe the experimental results nicely as can be seen, for example, in Figure 4 in the case of Zn_x_Fe_3−x_O_4_ nanoparticle pellets with x = 0 and x = 0.2. The *b* parameters are near the same as those determined from magnetic measurements according to relation (1). This fact stresses that really surface magnetization is involved in the ITMR process. The magnetoresistance in Fe_3_O_4_ pellets was shown to be rather low, behavior attributed to the damaged surface [46]. The negative polarization of the Fermi edge region (−30% to −40%) suggests that surface imperfections reduce the overall polarization by approximately 60% in Fe_3_O_4_ (001) thin films [64].

The *c* parameters are of the order of 10^−3^ T^−1^ and very close to those determined in double perovskites [61,62]-Table 2. The polarization of the nanocrystalline pellets increases with decreasing temperature.

Higher tunneling magnetoresistance has been generally observed in the surface-functionalized Fe_3_O_4_ [33,46,51,52,58]. The tunneling magnetoresistances increase as the surface is restored, as shown in oleic-acid coated [33,52,65,66], polystyrene coated [58], and pyridine coated Fe_3_O_4_ nanoparticles [67]. As in the case of bare ferrites, the magnetoresistances are well described by the relation (4) with the parameters *P*, *b,* and *c*, listed in Table 2. The determined polarization is higher than in bare nanocrystalline pellets. The *b* parameters describing the field dependences of the surface magnetizations are the same as those determined in bare nanoparticles or obtained from magnetic measurements. The *c* parameter, taking into account the spin disorder inside the grains, is generally higher than in the case of non-functionalized samples and depends on the applied pressure for obtaining pellets. The polarization also depends on measuring temperature. The spin polarization, P, determined in Fe_3_O_4_ based nanoparticles above the Vervey temperature, is somewhat lower than in double perovskites.

The coating material at the nanograin surface contributes additionally to the linear field dependence of magnetoresistivity. Probably, it is the result of the interface chemical bonding formed by the coordination of some elements (nitrogen, oxygen) of coating materials with the Fe ions at the surface, as already mentioned. Table 2 also gives the results of the analysis of magnetoresistance in some half-metallic double perovskites. The previous studies on both ball-milled Fe_3_O_4_ [50] as well as microcrystalline Sr-based double perovskites having dimensions around 1 µm [59,62,63] show that the field dependence of magnetization in describing ITMR must be that characteristic in the region close to grain boundary and not the bulk magnetization.

The spin-glass state, due to surface effects in double perovskites having weak anisotropy, is well described by 1/*H*^−1/2^ law [61,62]. Values *b* = 0.13 for Sr_2_FeMoO_6_ and *b* = 0.16 for Sr_2_FeMo_0.7_W_0.3_O_6_ were determined. The behavior of Fe_3_O_4_ magnetoresistances-based nanoparticle pellets is similar to that of double perovskites.

## 4. Conclusions

The magnetic properties of Fe_3_O_4_ - based nanoparticles are well described in a core–shell model, where the core is ferrimagnetically ordered, and the shell shows a spin glass type behavior. The spin-glass state is due to a fraction of magnetic ions located in the shell having one lattice parameter width and connected with the symmetry breaking of the surface structure. The reduced magnetization of the spin-glass component follows a field dependence *m_g_* = (1 − *b/H*^−1/2^), where the *b* parameters decrease linearly as the exchange interactions between the two sublattices increase.

The field dependences of Fe_3_O_4_-based nanoparticles magnetoresistances, experimentally determined, are well described if instead of the reduced magnetization of core, commonly used, that of the shell, of spin glass type is considered. Identical trends for the approach to saturation of reduced magnetizations as described by the *b* parameter are shown, both starting from magnetic measurements and magnetoresistances studies. Thus, for Fe_3_O_4_ nanoparticles, a value *b* ≅ 0.108 T^−1/2^ was obtained from magnetic measurements, while for magnetoresistances of the corresponding pellets, these are in the range *b* = (0.10–0.12)T^−1/2^. In case of Zn_0.18_Fe_2.82_O_4_ nanoparticles, the determined *b* = 0.174 T^−1/2^ from magnetization isotherm at T = 4 K is nearly the same as obtained from the analysis of field dependence of magnetoresistances in Zn_0.2_Fe_2.80_O_4_ nanoparticles pellets.

The magnetoresistances of Fe_3_O_4_—based nano-particles pellets are similar to those of A_2_FeMoO_6_ (A = Sr, Ba) double perovskites having grain sizes of ≅ 1 µm. The spin polarization, P, determined in Fe_3_O_4_ based nanoparticles above the Vervey temperature, are somewhat lower than in double perovskites. The crystal sizes and shell surfaces are different, influencing the polarization degree.

## Figures and Tables

**Table 1:** Lattice parameters and nanocrystallite sizes.

Sample	Lattice Parameter (nm)	Mean Crystallite Size (nm)
Fe_3_O_4_	0.8334(2)	17(1)
CoFe_2_O_4_ (4)	0.8379(1)	14.2(2)
Zn_0.12_Fe_2.88_O_4_	0.841(2)	27(2)
Zn_0.18_Fe_2.82_O_4_	0.842(3)	15(1)

**Table 2:** Data obtained from magnetoresistance measurements.

Nanoparticles Pellet	*T* (K)	*b* (T^−1/2^)	*c* (T^−1^)	*-P* (%)	Reference
Fe_3_O_4_ *d* = 20 nm	300	0.11	0.0027	13.4	[50]
Fe_3_O_4_ *d* = 8.9 nm	300	0.115	0.0026	17.6	[54]
Fe_3_O_4_ *d* = 20 nm	200	0.12	0.0045	24.2	[50]
Fe_3_O_4_ *d* = 8.9 nm	200	0.12	0.00265	27	[54]
Fe_3_O_4_ *d* = 10(2) nm	115	0.12	0.08	20.6	[64]
Fe_3_O_4_ *d* = 10(2) nm	115	0.12	0.078	33.4	[33]
Fe_3_O_4_ *d* = 30 nm	100	0.11	0.003	41.2	[38]
Fe_3_O_4_ amine monolayer *d* = 8 nm (sphere)	300	0.10	0.055	36.5	[46]
Fe_3_O_4_ amine monolayer *d* = 8 nm, (octahedra)	300	0.15	0.060	56	[46]
Fe_3_O_4_ *d* = 10.3 nm, polystyrene coated	280	0.10	0.008	39.4	[58]
Fe_3_O_4_ *d* = 10(2) nm, oleic acid coated	115	0.12	0.07	47.3	[33]
Fe_3_O_4_ *d* = 10–30 nm, polystyrene coated	110	0.15	0.013	56.8	[58]
Fe_3_O_4_ three-dimensional array	100	0.16	0.0024	38.5	[51]
Zn_0.2_Fe_2.8_O_4_ *d* = 30 nm	110	0.17	0.0044	30.5	[38]
Sr_2_FeMo_0.7_W_0.3_O_6_, perovskite	10	0.16	0.005	50	[62]
(Ba_0.8_ Sr_0.2_)_2_FeMoO_6_, perovskite	200	0.20	0.0017	60	[61]

## Data Availability

Not applicable.
